# Theoretical Orientations and the Stereotype Content Model – Are Prejudices Barriers to Psychotherapy Integration?

**DOI:** 10.32872/cpe.17975

**Published:** 2026-02-27

**Authors:** Johanna Schröder, Sebastian Trautmann, Nils F. Töpfer, Julian A. Rubel, Katinka Schweizer, Björn E. Hermans, Meike Shedden Mora, Mathias Kauff

**Affiliations:** 1 Institute for Clinical Psychology and Psychotherapy, Department of Psychology, Medical School Hamburg, Hamburg, Germany; 2 School of Human Sciences, Department of Psychotherapy Research and Clinical Psychology, Osnabrueck University, Osnabrueck, Germany; 3 Institute for Environmental, Social and Work Psychology, Department of Psychology, Medical School Hamburg, Hamburg, Germany; Philipps-University of Marburg, Marburg, Germany

**Keywords:** psychotherapy integration, stereotype content model, theoretical orientation, in-group, prejudices

## Abstract

**Background:**

Despite efforts to integrate psychotherapy, the field remains fragmented into distinct theoretical orientations and practical approaches. Prejudices held by psychotherapists towards those from other theoretical orientations may hinder cooperation in research and clinical practice. This study examines stereotypes among psychotherapists from different theoretical orientations and practical approaches (‘psychotherapy schools’) towards their in-group and out-group colleagues.

**Method:**

The cross-sectional online study assessed socially shared evaluations of ‘warmth’ and ‘competence’ from the stereotype content model in a sample of 586 German psychotherapists (60.9% licensed; 39.1% in training) from different psychotherapy schools (34.5% psychoanalytic and psychodynamic psychotherapists, 19.8% psychodynamic psychotherapists, 28.7% cognitive behavioural therapists, and 17.1% systemic therapists). Linear mixed-effects models were used to examine differences in evaluations based on the rater’s and the rated psychotherapy school.

**Results:**

The psychotherapists' assumed socially shared evaluations of ‘warmth’ and ‘competence’ varied depending on their psychotherapy school affiliation, with significantly higher evaluations assigned to their in-groups than to their out-groups.

**Conclusion:**

The results indicate in-group biases in the social perception of psychotherapists with different theoretical orientations, representing potential barriers to inter-group contact and collaboration. Addressing prejudices is key to strengthening integrative competence in both research and clinical practice.

## Highlights

- The discipline of psychotherapy is fragmented into different theoretical orientations.
- Psychotherapy integration approaches aim to strengthen effectiveness of psychotherapy.
- In-group biases affect social perceptions of psychotherapists regarding theoretical orientations.
- Addressing stereotypes may reduce prejudices and stimulate collaboration and integration.

In many countries, psychological interventions are considered a collection of independent treatment approaches, characterized by their own theoretical framework, concepts, and treatment foci (Rief et al., 2024). This division is not only evident in the way the different approaches are, in some countries, structured within health care systems, professional training programs, and licensing requirements, but also in the distinct terminologies or language used, which may hinder psychotherapists from recognizing similarities and points of complementarity (Goldfried, 2019; Rief et al., 2024). Ascribing to a specific theoretical orientation has long been an important identifying mark for many psychotherapists (Feixas & Botella, 2004). However, in the view of a growing group of researchers, this fragmentation keeps the discipline of psychotherapy in a pre-paradigmatic (Goldfried, 2019) or immature (Gaines et al., 2021) state. Decades ago, a movement for psychotherapy integration evolved, aiming “to reduce perpetuating the myth of separatism“ (Halgin, 1985), and to develop a framework for dialogue among psychotherapy schools (Feixas & Botella, 2004), resulting in an evidence-informed framework of valuable elements in various theories (Wakefield et al., 2020). Several approaches have been proposed as pathways to psychotherapy integration (Norcross, 1992): a) technical eclecticism (i.e., selecting most appropriate interventions for a specific person and symptom), b) theoretical integration (i.e., combining theories and applying their associated techniques), c) assimilative integration (i.e., grounding therapy in one theory while incorporating techniques drawn from other theoretical orientations), d) the “common factors” model (i.e., focusing on core elements contributing to effective psychotherapy, transcending specific theoretical orientations; Brown, 2015), and e) unification (i.e., applying coherent meta-theoretical frameworks for understanding how psychotherapy works; Marquis et al., 2021). Despite these efforts, fragmentation in the field of psychotherapy remains the rule rather than the exception, with Gaines et al. (2021) arguing that this lack of consensus is largely due to power struggles among competing psychotherapy schools. While other factors, such as historical development or institutional structures, may also contribute, it seems important to shed light on inter-group dynamics.

Social psychology provides a theory for understanding these dynamics: Research shows that individuals often rely on stereotypes, which are simplified and overgeneralized beliefs, about groups of individuals when evaluating them (Kanahara, 2006). According to Fiske (2012), stereotypes are patterns of perception linked to a group's societal interdependence and status. Negative stereotypes and, as a consequence, prejudices occur when individuals see themselves as belonging to one group, their in-group, which they perceive to be distinct from and superior to other groups, their out-groups (Dovidio & Gaertner, 1993; Tajfel & Turner, 1979). Stereotypes play a key role in reducing intergroup contact and cooperation (e.g., Stephan & Renfro, 2002). The stereotype content model (SCM; Fiske et al., 2002) provides a functional explanation for why stereotype content is organized into two dimensions: It suggests that the degree of ‘warmth’ and ‘competence’ is determined by societal status and perceived competitiveness. Along the warmth dimension, out-groups differ in the extent to which they are perceived as intending to harm or threaten the in-group. Along the competence dimension, out-groups differ in the extent to which they are perceived as capable of implementing their goals, e.g., to harm the in-group (Eckes, 2002). The in-group is typically seen as warm and competent, while out-groups are often perceived as lacking in either warmth or competence (Fiske et al., 2002). The mixed-stereotypes hypothesis proposes that many stereotypes combine low ratings on one dimension with high ratings on the other (Eckes, 2002). Building on social comparison-based and attributional models of emotion, the resulting stereotype clusters are associated with four unique emotional responses: admiration, contempt, envy, and pity (Cuddy et al., 2008; Fiske et al., 2002). Paternalistic stereotypes (high warmth, low competence) are associated with groups such as elderly or disabled people, envious stereotypes (low warmth, high competence) are associated with groups such as wealthy people, admiration stereotypes (high warmth, high competence) are applied to the in-group or close allies, and contemptuous stereotypes (low warmth, low competence) are associated with groups such as poor people or welfare recipients.

In many countries, professional training in psychotherapy remains largely focused on specific techniques from a single school (Rief, 2021). This training, which spans several years, includes theoretical and practical education, intervision, and supervision, fostering in-group socialization and strong identification with a particular theoretical orientation. In Germany, four psychotherapy schools are recognized by national health insurance, each with distinct theoretical and practical approaches: Psychoanalytic Psychotherapy (PA) focuses on resolving unconscious processes stemming from early life experiences, alleviating patients’ symptoms by helping to understand and transform unexplained emotional experiences (Rudolf et al., 2012). Psychodynamic Therapy (PDT) builds on the same etiological conception, although it applies a more structured approach, focusing on unconscious processes influencing current symptoms, aiming to increase self-awareness and promote emotional change (Leichsenring et al., 2023). Cognitive Behaviour Therapy (CBT) is a structured and goal-oriented approach aiming to identify and modify maladaptive thoughts and behaviors contributing to psychological distress (Davis et al., 2017), and to improve interpersonal, problem solving and emotion regulation skills (Ford, 2017). Systemic Therapy (ST) focuses on circular interaction patterns within social systems and systematically incorporates multi-person settings. It emphasizes a non-pathologizing approach, highlighting patients’ resources and focusing on solutions rather than on problems (Lorås et al., 2017).

Grounded on the SCM, we hypothesize that psychotherapists trained in different psychotherapy schools (PA, PDT, CBT, ST) evaluate psychotherapists of their in-groups higher in ‘warmth’ (H1) and ‘competence’ (H2) than their three out-groups.

## Method

### Participants and Recruitment

The study included German psychotherapists with a psychotherapeutic practice license in PA, PDT, CBT, or ST or psychotherapists in training for one of these licenses. A power analysis for an independent *t*-test with Stata 15.1, based on a standardized mean difference between groups of *f*^2^ = .25, resulted in a target sample size of 50 participants per group, totaling at least 200 participants, for ensuring that the 95% confidence interval would not include *f*^2^ = .05, which we defined as the threshold for a meaningful difference. As the survey posed minimal risk and effort for the participants, we aimed to recruit the highest possible number of participants within a six-month time frame (January to June 2024) to enhance generalizability of the results. Participants were recruited through mailing lists of several German psychotherapy associations, inpatient and outpatient clinics, clinical psychology departments at universities, and psychotherapist training centers. As optional compensation, 20 vouchers à 50 € for a social online bookstore were offered, with a total value of 1,000 €.

### Study Design, Procedure and Material

The current study used a descriptive cross-sectional design. The anonymous online study was programmed with unipark (Tivian XI GmbH, 2025). After giving informed consent to participate in the study, the participants started the online survey. They were asked whether they are licensed or in training for a license in PA, PDT, CBT, or ST. Selecting “not applicable” violated the inclusion criterion and led to the termination of the online survey. The survey measured the participants’ perceptions of consensual in-group evaluations of warmth and competence towards their own psychotherapy school (in-group) as well as their attributions of warmth and competence to the respective other three psychotherapy schools (out-groups). The wording of the question was: “How competent/warm do most psychotherapists of your psychotherapy school perceive psychotherapists of PA / PDT / CBT / ST?". The items were adapted from previous studies testing assumptions related to the SCM (e.g., Asbrock, 2010; Cuddy et al., 2009; Fiske et al., 2002). In line with these studies, we measured consensual stereotypes, referring to socially shared evaluations of warmth and competence rather than individual attitudes, to reduce social desirability bias (Kotzur et al., 2020). The response options of these items ranged from 1 – not at all, 2 – rather less, 3 – moderately, 4 – rather more, 5 – very much. Additionally, the survey included further questions regarding participants’ professional practice and attitudes in the field of psychotherapy, which are related to other research questions (see study registration).

### Data Analysis

In the data cleaning process, 19 cases were excluded from the dataset of 605 participants, as they reported being trained in multiple psychotherapy schools, resulting in a sample of *N* = 586. As most of the PA therapists were also trained in PDT (*n* = 193, 95.5%) and nine (4.5%) PA therapists were solely trained in PA, these groups were united in the group called PA, whereas the PDT group was solely trained in PDT. The dataset included 2,333 warmth ratings and 2,335 competence ratings at level one (intra-individual variance) and 586 subjects at level two (inter-individual variance). Eleven warmth ratings and nine competence ratings were missing due to unintended missing mandatory response settings in the online survey. In the analysis, missing data were handled by excluding rows with missing values from the model fitting process, ensuring that the dataset's structure is maintained for prediction and residual checks. Dummy variables were used to represent the categorical predictors (own school, rated school, and their interaction). To examine whether the evaluations of psychotherapy schools (independent variable; PA, PDT, CBT, ST) vary based on the rater’s own psychotherapy school (independent variable; PA, PDT, CBT, ST), we conducted two linear mixed-effects models (LMM): one for ‘warmth’ evaluations and one for ‘competence’ evaluations (dependent variables). In both models, fixed effects were specified for the rated psychotherapy school and the rater’s own psychotherapy school, each with four categories (PA, PDT, CBT, and ST). Although the pre-registered analysis plan aimed for MANOVAs, we adjusted the statistical approach favouring LMM. These changes were implemented to better align with the data and improve the robustness of the findings. We tested a random intercept model for each subject to account for possible within-subject correlations due to repeated ratings and a model with a random slope for the rated psychotherapy school to capture possible individual differences in the perception of the psychotherapy schools. The inclusion of a random intercept and random slopes for the rated psychotherapy schools resulted in an improved model fit compared to simpler models. The LMMs were estimated using the lme4 package (Bates et al., 2015), and model fit was evaluated using marginal and conditional *R*^2^ values calculated with the MuMIn package (Barton & Barton, 2015) in *R*. Model assumptions (e.g., normality, homoscedasticity) were checked visually using residual plots, and model fit was evaluated via the Akaike Information Criterion (AIC) and log-likelihood ratio tests. The Intraclass Correlation Coefficient (ICC) was calculated to assess the proportion of variance in the outcome variable attributable to differences between individuals. All statistical analyses were conducted with a significance threshold of *p* = .05.

## Results

### Sample Characteristics

Table 1 shows the sample characteristics of the participants in the current study.

**Table 1:** Sample *C*haracteristics (N = 586)

Age in years		
*M* (*SD*), *Mdn*	42.3 (12.3)	39
Range	23 – 80	
Sex	*n*	%
Female	430	73.4
Male	154	26.3
Diverse	2	0.3
Professional status		
Licensed psychotherapist – *n*, %	357	60.9
Years with license – *M (SD), Mdn*	10.6 (9.4)	7
Psychotherapist in training – *n*, %	229	39.1
Years in training – *M (SD), Mdn*	3.2 (2.6)	3
Specialization^a^	*n*	%
Psychotherapy for adults	493	84.1
Psychotherapy for children and youths	132	22.5
Neuropsychological Psychotherapy	6	1.0
Field of university degree	*n*	%
Psychology	490	83.6
Medicine	35	6.0
Social pedagogy and social work	37	6.3
Pedagogy	32	5.5
School of psychotherapy	*n*	%
PA or PA & PDT	202	34.5
PDT (without PA)	116	19.8
CBT	168	28.7
ST	100	17.1
Professional focus^a^	*n*	%
Practicing psychotherapy	562	95.9
Research	72	12.3
Teaching	97	16.6
Work setting^a^	*n*	%
Private practice	302	51.5
Psychotherapy training center	146	24.9
Psychiatry and psychotherapy inpatient clinic	97	16.6
University	63	10.8
Inpatient clinic for psychosomatic medicine	39	6.7
Inpatient clinic for rehabilitation care	31	5.3
Psychotherapy outpatient clinic	31	5.3
Medical care center	15	2.6
Other setting	104	17.7

*Note. M* = Mean; *SD* = Standard Deviation; *Mdn* = Median; PA = Psychoanalytic Psychotherapy (PA: *n* = 193, PA and PDT: *n* = 9 combined); PDT = Psychodynamic Psychotherapy; CBT = Cognitive Behaviour Therapy; ST = Systemic Therapy.

^a^Multiple answers are possible.

### Effects of Psychotherapy Schools on Socially Shared Evaluations of Warmth and Competence

Significant interaction effects between the rated psychotherapy school and the rater’s psychotherapy school were found for all interaction terms regarding warmth as well as competence, with large effect sizes (see Table 2). This suggests that the assumed socially shared evaluations of warmth and competence toward out-groups were influenced by the participants’ psychotherapy school affiliation, supporting the in-group bias proposed in H1 and H2.

**Table 2:** Results of Linear Mixed Model With the Rater’s Psychotherapy School and the Rated Psychotherapy School as Fixed Effects and Warmth and Competence Ratings as Dependent Variables

Fixed Effects	Warmth					Competence				
	*B*	*SE*	95% CI	*t*(*df*)	*p*	*B*	*SE*	95% CI	*t*(*df*)	*p*
(Intercept)	3.693	0.064	[3.568, 3.817]	57.887 (1735)	< .001***	4.476	0.062	[4.356, 4.597]	72.642 (1737)	< .001***
Rated psychotherapy school PDT	0.172	0.064	[0.046, 0.298]	2.673 (1735)	.008**	-0.523	0.058	[-0.637, -0.410]	-9.019 (1737)	< .001***
Rated psychotherapy school CBT	-1.010	0.085	[-1.176, -0.843]	-11.857 (1735)	< .001***	-1.486	0.082	[-1.646, -1.325]	-18.093 (1737)	< .001***
Rated psychotherapy school ST	-0.364	0.085	[-0.529, -0.199]	-4.304 (1735)	< .001***	-1.214	0.074	[-1.358, -1.070]	-16.497 (1737)	< .001***
Rater’s psychotherapy school PDT	-0.667	0.105	[-0.873, -0.461]	-6.339 (582)	< .001***	-0.373	0.102	[-0.572, -0.174]	-3.668 (582)	< .001***
Rater’s psychotherapy school CBT	-1.669	0.094	[-1.853, -1.484]	-17.692 (582)	< .001***	-1.589	0.091	[-1.768, -1.411]	-17.438 (582)	< .001***
Rater’s psychotherapy school ST	-1.513	0.110	[-1.729, -1.297]	-13.705 (582)	< .001***	-1.246	0.107	[-1.455, -1.037]	-11.685 (582)	< .001***
PDT-PDT	0.871	0.106	[0.663, 1.079]	8.197 (1735)	< .001***	0.592	0.096	[0.405, 0.779]	6.191 (1737)	< .001***
PDT-CBT	0.846	0.141	[0.571, 1.121]	6.013 (1735)	< .001***	0.658	0.136	[0.393, 0.923]	4.856 (1737)	< .001***
PDT-ST	0.848	0.140	[0.575, 1.121]	6.072 (1735)	< .001***	0.464	0.121	[0.227, 0.702]	3.826 (1737)	< .001***
CBT-PDT	1.161	0.095	[0.975, 1.347]	12.205 (1735)	< .001***	1.279	0.086	[1.112, 1.447]	14.908 (1737)	< .001***
CBT-CBT	2.597	0.126	[2.350, 2.844]	20.571 (1735)	< .001***	2.819	0.122	[2.582, 3.057]	23.191 (1737)	< .001***
CBT-ST	2.168	0.125	[1.923, 2.412]	17.319 (1735)	< .001***	2.104	0.109	[1.891, 2.316]	19.312 (1737)	< .001***
ST-PDT	0.898	0.111	[0.680, 1.115]	8.066 (1735)	< .001***	1.063	0.100	[0.867, 1.260]	10.591 (1737)	< .001***
ST-CBT	1.710	0.148	[1.421, 1.998]	11.583 (1735)	< .001***	1.936	0.142	[1.658, 2.214]	13.607 (1737)	< .001***
ST-ST	2.476	0.147	[2.189, 2.762]	16.884 (1735)	< .001***	2.231	0.128	[1.982, 2.480]	17.495 (1737)	< .001***

*Note.* By-subject and by rated psychotherapy school random slopes were included for all fixed effects. PA = Psychoanalytic Psychotherapy; PDT = Psychodynamic Psychotherapy; CBT = Cognitive Behaviour Therapy; ST = Systemic Therapy. In the last nine lines, the first-named school represents the rater’s school and the second one the rated school. *SE* = Standard Error; CI = Confidence Interval. The intercept represents the average warmth/competence rating by PA psychotherapists to their in-group, as PA serves as the reference category for both fixed effects.

*p < .05. **p < .01. ***p < .001.

In the warmth model, the marginal *R*^2^, which represents the proportion of variance explained by the fixed effects alone, was $R_{m}^{2}$ = 0.360. The conditional *R*^2^, representing the variance explained by both the fixed and random effects, was $R_{c}^{2}$ = 0.865. The ICC was 0.583, suggesting that 58% of the total variance in the outcome variable is attributable to between-subject variability, while the remaining 42% is due to within-subject differences.

In the competence model, the marginal *R*^2^, which represents the proportion of variance explained by the fixed effects alone, was $R_{m}^{2}$ = 0.265. The conditional *R*^2^, representing the variance explained by both the fixed and random effects, was $R_{c}^{2}$ = 0.866. The ICC was 0.694, suggesting that 69% of the total variance in the outcome variable is attributable to between-subject variability, while the remaining 31% is due to within-subject differences.

**Figure 1:**
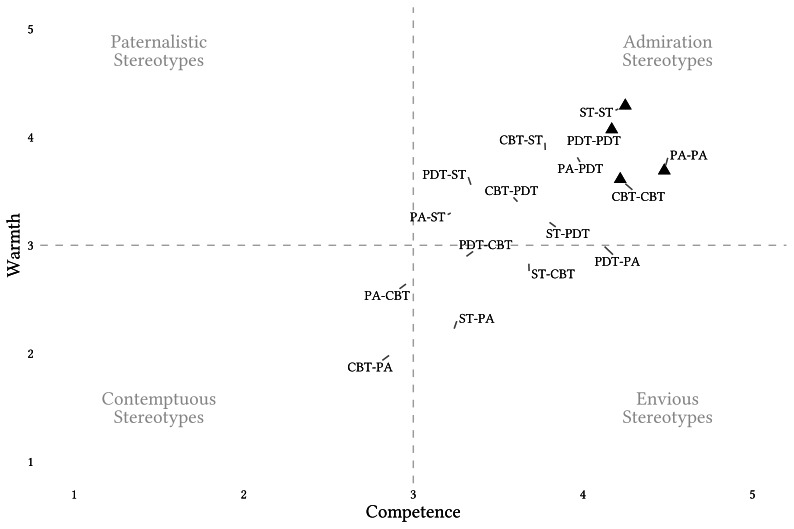
Estimated Marginal Means and Confidence Intervals From a Linear Mixed Model Assessing Assumed Shared Evaluations of Warmth and Competence Between Four Psychotherapy Schools *Note.* PA = Psychoanalytic Psychotherapy; PDT = Psychodynamic Psychotherapy; CBT = Cognitive Behaviour Therapy; ST = Systemic Therapy. The first-named school represents the rater’s school and the second one the rated school. Paternalistic Stereotypes: high warmth, low competence; Envious Stereotypes: low warmth, high competence; Admiration Stereotypes: high warmth, high competence; Contemptuous Stereotypes: low warmth, low competence (Fiske et al., 2002).

**Figure 2:**
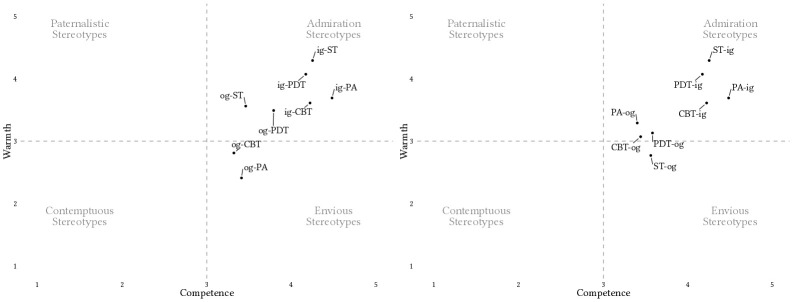
EMM and Confidence Intervals From Linear Mixed Models Assessing Assumed Shared Evaluations of Warmth and Competence Between Four Psychotherapy Schools *Note.* Left plot: Assumed shared in-group and out-group evaluations regarding the four psychotherapy schools. Right plot: Assumed shared evaluations by the psychotherapists of the four schools of their in-groups and their out-groups. PA = Psychoanalytic Psychotherapy; PDT = Psychodynamic Psychotherapy; CBT = Cognitive Behaviour Therapy; ST = Systemic Therapy. ‘ig’ represents the estimated marginal mean (EMM) of the respective ingroup, ‘og’ represents the average EMM of the respective outgroups. The first elements in the labels represent the *rater’s* psychotherapy school, ingroup or outgroup and the second elements represent the *rated* psychotherapy school, in-group or out-group. Paternalistic Stereotypes: high warmth, low competence; Envious Stereotypes: low warmth, high competence; Admiration Stereotypes: high warmth, high competence; Contemptuous Stereotypes: low warmth, low competence (Fiske et al., 2002).

Figure 1 illustrates the combined estimated marginal means (EMM) from both LMM, one using warmth ratings and the other using competence ratings as outcome variables. For the numeric details we refer to the Supplementary Materials (Schröder et al., 2026S-a). Most convergence points are concentrated in the ‘admiration’ quadrant. A pattern of ‘envious’ stereotypes is evident in PDT and ST’s evaluations of CBT, as well as in ST’s evaluations of PA. A pattern of ‘contemptuous’ stereotypes can be observed in PA’s evaluations of CBT and CBT’s evaluations of PA. The findings show no ‘paternalistic’ stereotypes among psychotherapy schools. Figure 2 illustrates the results grouped by in- and out-groups.

## Discussion

The current study investigated in-group biases among psychotherapists from different theoretical orientations (PA, PDT, CBT, ST). The findings show that psychotherapists of all four schools rated their in-group significantly higher in warmth and competence than their out-groups, supporting both hypotheses. This in-group bias mirrors patterns observed in general populations, where in-groups are typically rated higher in both dimensions across various social categories, though such patterns have primarily been seen in Western cultures (Cuddy et al., 2009; Fiske et al., 2002). The results align with Larsson et al. (2013), who found that psychotherapists misjudge the attitudes of psychotherapists with other theoretical orientations.

The current study found reciprocal ‘admiration’ stereotypes in the evaluations between PA and PDT therapists, which can not only be explained by the shared epistemic framework, but also by the fact that most PA therapists were also licensed in PDT. ST therapists assumed shared ‘envious’ stereotypes towards their out-groups, which might be explained by their relatively recent approval for reimbursement within Germany’s statutory health insurance system since 2019. While this official recognition underscores the growing status of ST, its training institutes and integration into university curricula are still limited compared to other psychotherapy schools. PA and CBT therapists tend to be associated with 'envious' stereotypes by their out-groups. This may be explained by the relatively high professional prestige of PA and CBT. The historic influence of PA extends beyond psychotherapy into philosophy and cultural discourse (Lear, 2003), while CBT is highly regarded for its strong empirical foundation (App & Dobson, 2010). The findings further indicate ‘contemptuous’ stereotypes between PA and CBT therapists. This can be explained by a “threat of otherness” (Govrin, 2016), which may be more pronounced between these two groups, compared to PDT or ST, when considering the pronounced distinctness in their theoretical orientations and practical approaches. However, drawing on prejudice research and a developmental approach based on attachment and identity theory (De Levita, 1965), a secure identity does not lead to higher scores of envy or prejudice towards “others”, but would rather allow for openness towards differences and pluralism. Thus, rigidity and envy towards other schools might be understood as potentially reflecting an insecure attachment and fragile identification, which could be pseudo-stabilized by devaluing other approaches instead of accepting or even appraising diversity for the sake of different groups of patients (see also Golec de Zavala et al., 2009). This interpretation remains speculative, and further empirical research would be needed to explore whether such dynamics occur within psychotherapy schools.

The current findings on stereotypes among psychotherapists from different theoretical orientations are relevant to approaches to psychotherapy integration, as in-group favoritism and prejudices towards out-groups may impede intergroup contact and collaboration. When aiming for cohesive frameworks for unified psychotherapy, advancing the discourse on common factors and developing reasonable integrative approaches, constructive cooperation between psychotherapists of different theoretical orientations is essential. Encouraging psychotherapists to adopt an open-minded and patient-oriented approach could help mitigate the limitations of rigid adherence to a single school and facilitate the development of efficient, personalized care. Discussing stereotypes among psychotherapists of different theoretical orientations in university teaching, professional training, interdisciplinary workshops, and conferences could provide opportunities for intergroup contact, enhancing mutual understanding. Dialogue about stereotypes may help to contextualize and deconstruct them, promoting a more nuanced perception of different therapeutic approaches. This could help focus on common grounds and goals, creating a shared professional identity as psychotherapists and paving the way for more effective psychotherapy integration. While psychotherapy integration offers promising avenues to further develop psychotherapy as an academic discipline, it also raises important challenges beyond in-group bias: In a study by Kaluzeviciute-Moreton and Lloyd (2024), psychotherapists expressed the belief that certain clinical techniques or modalities are theoretically and politically incompatible. For instance, some cognitive behavioural therapists view psychoanalysis as less adaptable, both to integrative approaches and to modern research. Conversely, psychoanalytic practitioners regard CBT as a dominant paradigm that is less open to integrating ideas and techniques from less evidence-based modalities. On a theoretical level, the interviewed psychotherapists highlighted the challenge of epistemic integration, noting the different philosophies underlying CBT and psychoanalysis. A unified framework, on the other hand, may risk oversimplifying theoretical differences, which are rooted in these distinct epistemic traditions. It becomes clear that integrative practice is disruptive, necessitating a broader understanding of various psychotherapeutic approaches to “find meaning and the right components” (Gordon et al., 2021). On another level, in some countries, like Germany, there are also concerns that psychotherapy integration could undermine the provision of psychotherapy under public health insurance schemes (Strauß, 2024). Additionally, a key obstacle may lie in misunderstandings surrounding the concept of psychotherapy integration or disagreements over which proposed pathways should be considered.

These challenges can only be overcome by trans-theoretical cooperation, constructive exchange, and critical reflection. At least, it should facilitate the recognition of the unique characteristics of each psychotherapy approach within its own underlying epistemic framework. Ultimately, acknowledging the diversity of approaches can, from a dialectic perspective, also be appreciated as a rich cultural resource that can be beneficial for the diversity of individuals in need of psychotherapy.

Several limitations should be considered when interpreting the results of the current study. First, the sample was not representative of the psychotherapist population in Germany or internationally, limiting generalizability. Further, the sample might be biased due to online assessment, although the sample shows great variance, especially regarding age and working years. As all authors were psychologists, recruitment resulted in a sample with only a few psychotherapists holding medical university degrees or being psychiatrists. Second, the SCM was originally developed as a universal measure of ‘shared cultural’ stereotypes, i.e., participants’ perceptions of what most individuals in a society think about a given target group. One experimental study suggests that response instruction (shared cultural perspective vs. individual perspective, i.e., what individuals personally think about the target group) does not significantly influence stereotype scale outcomes (Findor et al., 2020). However, this effect may depend on the specific target group and has not been evaluated for the population at hand. Consequently, using the shared cultural perspective in the current study may have led to an exaggeration of stereotypes, which should be considered in the interpretation of the results. On the other hand, the shared cultural perspective may have helped reduce a social desirability bias, potentially preventing the understatement of stereotypes. It would be worthwhile to incorporate both perspectives in future studies. The use of multilevel modeling has proven to be crucial in capturing the variability in psychotherapists’ evaluations at both the individual and group levels. The high ICC values underscore the significant between-individual variability, and substantial differences between marginal and conditional *R*^2^ underscore the importance of accounting for individual differences, captured by the random effects structure in the model. This suggests that personal experiences or individual biases may play a significant role in stereotype formation and expression.

The study results raise important questions for future research. First, including further theoretical orientations in international contexts, such as humanistic psychotherapy (Schneider & Leitner, 2002), could provide a broader understanding of how stereotypes vary across cultural and educational contexts. Second, investigating how stereotypes of psychotherapists with different therapeutic orientations relate to other dimensions of stereotype content, e.g., the Agency, Beliefs, Communion (ABC) model (Koch et al., 2016), may yield valuable insights. Third, investigating the role of individual factors such as gender, clinical experience, academic background, and experiences with interdisciplinary work could shed light on the mechanisms underlying stereotype formation. Fourth, for a better understanding of the origin of latent stereotypes in psychotherapists, it would be worth examining the quality and security of affiliation and the processes of attaining a (specific) psychotherapeutic identity. Moreover, it could be valuable to investigate the role of perceived intergroup threat in stereotype formation (Stephan et al., 2009) – particularly the influence of realistic threats (e.g., concerns about losing resources to another psychotherapy approach) and symbolic threats (e.g., concerns about losing normative dominance).

The study findings demonstrate a stereotype-associated in-group bias between psychotherapists of different theoretical orientations. Addressing these stereotypes could help reduce prejudices and stimulate collaboration among psychotherapists of different theoretical orientations, allowing the discipline of psychotherapy to move toward a more integrative paradigm, while still valuing the diversity of established approaches.

## Supplementary Materials

The Supplementary Materials contain the following items:

- *Preregistration* (Schröder et al., 2024S)
- *Supplementary File 1* (Schröder et al., 2026S-a): This supplement includes a table summarizing the estimated marginal means of the results of the linear mixed models assessing warmth and competence evaluations between psychotherapists of four psychotherapy approaches. The plots in Figures 1 and 2 are based on the statistics presented in this table.
- *Supplementary File 2* (Schröder et al., 2026S-b): This supplement contains the data file.

SchröderJ.
Shedden MoraM.
KauffM.
HermansB. E.
SchweizerK.
TöpferN. F.
TrautmannS.
 (2024S). Attitudes towards different theoretical orientations among psychotherapists
[Preregistration]. PsychOpen. https://osf.io/px96t


SchröderJ.
TrautmannS.
TöpferN. F.
RubelJ. A.
SchweizerK.
HermansB. E.
Shedden MoraM.
KauffM.
 (2026S-a). Supplementary materials to "Theoretical orientations and the stereotype content model – Are prejudices barriers to psychotherapy integration?"
[Additional table]. PsychOpen. 10.23668/psycharchives.21575
PMC1295530441783469

SchröderJ.
TrautmannS.
TöpferN. F.
RubelJ. A.
SchweizerK.
HermansB. E.
Shedden MoraM.
KauffM.
 (2026S-b). Supplementary materials to "Theoretical orientations and the stereotype content model – Are prejudices barriers to psychotherapy integration?"
[Research data and codebook]. PsychOpen. 10.23668/psycharchives.21574
PMC1295530441783469

## Data Availability

The data is available in the Supplementary Materials (Schröder et al., 2026S-b).

## Preregistration

The study was preregistered in the Open Science Framework (https://osf.io/px96t).

## Reporting Guidelines

The manuscript was written according to the JARS-Quant reporting standards.

## Social Media Accounts

*Johanna Schröder:* LinkedIn, Bluesky; *Katinka Schweizer:* LinkedIn; *Nils F. Töpfer:* LinkedIn, Bluesky; *Meike Shedden Mora:* Bluesky, *Mathias Kauff:* Bluesky
